# Internalized Stigma, Insight, Social Anxiety and Quality of Life in First-Episode Psychosis: A Cross-Sectional Study

**DOI:** 10.3390/healthcare14152342

**Published:** 2026-08-01

**Authors:** Eugenia I. Toki, Andreas Karampas, Georgios Markozannes, Iliana Ntourou, Alexandros-Georgios Asimakopoulos, Evangelos Ntouros, Marios Plakoutsis, Georgios Georgiou, Konstantinos Kotsis, Sofia Petrakou, Vassiliki Siafaka, Petros Petrikis

**Affiliations:** 1 Department of Speech and Language Therapy, School of Health Sciences, University of Ioannina, 45500 Ioannina, Greece; toki@uoi.gr (E.I.T.); siafaka@uoi.gr (V.S.); 2 Department of Psychiatry, University of Ioannina School of Medicine, 45110 Ioannina, Greece; an.karampas@gmail.com (A.K.); dourouiliana@gmail.com (I.N.); marios.plak@gmail.com (M.P.); ggeorg1987@hotmail.com (G.G.); konkotsis@uoi.gr (K.K.); s.petrakou@gmail.com (S.P.); 3 Department of Hygiene and Epidemiology, University of Ioannina School of Medicine, 45110 Ioannina, Greecea.asimakopoulos@uoi.gr (A.-G.A.); 4 2nd Department of Psychiatry, School of Medicine, Aristotle University of Thessaloniki, 54124 Thessaloniki, Greece; durogoo8@gmail.com

**Keywords:** psychotic disorders, stigma, social, quality of life, social anxiety, insight

## Abstract

**Background:** People with schizophrenia are one of the most stigmatized social groups. The purpose of this study was to assess stigma, quality of life, social anxiety and illness insight in a sample of first-episode psychosis (FEP) patients and to investigate possible correlations between these variables and psychopathology/socio-demographic factors of the study group. **Methods:** This observational cross-sectional study included 90 patients (55 males, 35 females) with a mean age of 31.8 ± 8.6 years (18–52) experiencing a first episode of psychosis. The mean ± SD duration of untreated psychosis was 13.4 ± 6.2 weeks. The medications used were risperidone (n = 24; 26.7%), clozapine (n = 34; 37.8%), and olanzapine (n = 32; 35.6%). The tools used for this study were the Internalized Stigma for Mental Illness Scale (ISMI), World Health Organization Quality of Life Assessment-BREF (WHOQoL-BREF), Liebowitz Social Anxiety Scale (LSAS-SR), Schedule for the Assessment of Insight—Expanded version (SAI-E), and Positive and Negative Syndrome Scale (PANSS). **Results:** The mean ISMI total score was 50.9 ± 7.9 (range: 29–84), with 56.6% of patients scoring greater than 50, indicative of internalized stigma. Mean PANSS total scores improved from 76.1 ± 7.2 at baseline to 44.9 ± 6.3 after six months of follow-up (*p* < 0.001). Patients demonstrated insight of (mean ± SD) 20.3 ± 4.5, measured by SAI-E, and moderate-to-good quality of life across WHOQOL-BREF domains. The results of this study indicated that the ISMI scale showed statistically significant linear correlations with the LSAS-SR [anxiety (r = 0.399, *p* < 0.001) and avoidance (r = 0.421, *p* < 0.001)], with positive correlations and approximately medium associations. In the multivariable analyses, ISMI was inversely associated with all WHOQoL-BREF domains (physical health: −0.72 units 95% CI: −1.09 to −0.35, *p* < 0.001; mental health:−1.04, 95% CI: −1.45 to −0.63, *p* < 0.001; social relationships: −0.79, 95% CI: −1.39 to −0.18, *p* = 0.012; environmental health: −0.70, 95% CI: −1.00 to −0.39, *p* < 0.001). Multivariable models were adjusted for age, gender, medication, marital status, having children, employment, residence, DUP, SAI-E, ISMI, and LSAS-SR. **Conclusions:** Stigma is positively correlated with social anxiety. Higher stigma levels were independently linearly associated with lower levels of quality of life, even after adjusting for other predictor variables. Our results also indicated an association between higher insight levels and elevated odds of high internalized stigma. Stigma in first-episode psychosis patients is negatively associated with quality of life and positively associated with social anxiety/avoidance.

## 1. Introduction

Stigma refers to a characteristic or attribute that is socially devalued and associated with negative stereotypes within a particular social context, resulting in negative attitudes and discriminatory behaviors toward the stigmatized individual [1]. Among people with severe mental illness, stigma is highly prevalent, with approximately 87% reporting experiences of stigmatization [2]. Individuals with schizophrenia are among the most stigmatized groups, frequently perceived as dangerous, unpredictable, and unlikely to recover [3,4]. Many patients describe stigma as more disabling than the illness itself, referring to it as a “second illness” [5].

Thornicroft (2007) [6] conceptualized stigma as comprising three interrelated psychological dimensions: knowledge (ignorance or misinformation), attitudes (prejudice), and behaviors (discrimination). From the perspective of the individual, stigma can further be categorized into perceived stigma, experienced stigma, and internalized stigma [7]. Perceived stigma refers to the belief that society holds negative views about a person’s condition. Experienced stigma encompasses direct discrimination, verbal or physical abuse, bullying, and reduced opportunities in education, employment, parenting, and interpersonal relationships because of the stigmatized attribute. Internalized stigma occurs when individuals accept and apply these negative societal stereotypes to themselves.

The consequences of stigma are profound and extend across multiple domains of illness and recovery. Internalized stigma has been associated with reduced adherence to antipsychotic medication, contributing to higher relapse rates, more frequent hospitalizations, and poorer clinical outcomes [8]. Individuals with psychosis have also reported concealing their medication and taking it in private to avoid being identified as having a mental illness [9]. Furthermore, stigma influences patients’ experiences throughout psychiatric hospitalization, including admission procedures, interactions with healthcare staff, and reintegration into the community following discharge [10]. Hospitalization-related stigma has also been identified as a potential risk factor for suicide attempts during the vulnerable post-discharge period [11].

Although stigma has been extensively investigated in chronic schizophrenia, relatively few studies have focused on individuals experiencing a first episode of psychosis (FEP) [12]. This population may be particularly vulnerable, as the onset of psychosis is often accompanied by the first contact with psychiatric services and the adoption of a psychiatric diagnosis, both of which may increase exposure to stigmatizing attitudes and stereotypes. At the same time, the early stage of illness offers a critical opportunity for anti-stigma interventions, which may be more effective before stigmatizing beliefs become entrenched [12]. Early intervention during the first years of treatment may help prevent the long-term negative consequences of diagnostic labeling and improve functional recovery [13]. Moreover, stigma has been shown to influence treatment engagement, social functioning following remission, and early clinical improvement in individuals with FEP [13,14].

People experiencing a first psychotic episode frequently report feelings of shame, social anxiety, and social exclusion, which may adversely affect their interpersonal relationships and community participation. Consistent with this, individuals with recent-onset psychosis exhibit higher levels of social anxiety and poorer quality of life than the general population [14,15]. In addition, although improving insight into illness is a primary goal of early-intervention services, greater insight may paradoxically increase vulnerability to internalized stigma by enhancing awareness of the negative stereotypes associated with psychotic disorders [16]. Understanding this relationship is essential to ensure that interventions aimed at improving insight do not inadvertently increase self-stigma.

Despite growing recognition of the importance of stigma in early psychosis, evidence examining the interplay between internalized stigma, insight, social anxiety, quality of life, and psychopathology remains limited. To our knowledge, this is the first study to investigate these factors simultaneously in a sample of individuals with first-episode psychosis. A better understanding of these relationships may identify modifiable therapeutic targets and inform the development of comprehensive early-intervention strategies aimed at improving both clinical and psychosocial outcomes.

The aim of the present study was to examine the relationships among internalized stigma, insight, social anxiety, quality of life, and psychopathology in individuals with first-episode psychosis.

## 2. Methods

This was an observational cross-sectional study conducted within a specialized Early Intervention in Psychosis service, assessing psychosocial outcomes six months following stabilization after a first psychotic episode.

### 2.1. Participants

Patients were recruited from the Early Intervention in Psychosis Unit, Department of Psychiatry, University Hospital of Ioannina, Greece, from October 2019 to June 2023. Ninety patients were enrolled in the study according to the following inclusion and exclusion criteria.

Inclusion criteria were: a. experience and recovery from a first episode of psychosis, defined as the first experience of psychotic symptoms (delusions, hallucinations, odd behavior) and the first contact with psychiatric health services [17]; b. diagnosis of brief psychotic episode, schizophreniform disorder or schizophrenia following the criteria of the Diagnostic and Statistical Manual of Mental Disorders-V (DSM-V) [18]) with the diagnostic assessment being clinician-assigned by an experienced psychiatrist; c. members of the outpatient early-intervention service for at least six months, and agreement to provide written informed consent.

Exclusion criteria were: a. history of alcohol and/or substance abuse, b. refusal to give written informed consent, and c. inability to understand and write in the Greek language for any reason.

Five patients were excluded from the study because they refused to give written informed consent and two because they were not fluent in Greek.

### 2.2. Materials and Methods

#### 2.2.1. Internalized Stigma for Mental Illness Scale

The Internalized Stigma for Mental Illness Scale (ISMI) is a self-report questionnaire comprising 29 items rated from 1 (strongly disagree) to 4 (strongly agree). ISMI comprises 5 subscales assessing alienation (items 1, 5, 8, 16, 17, 21), stereotype endorsement (items 2, 6, 10, 18, 19, 23, 29), discrimination experience (items 3, 15, 22, 25, 28), social withdrawal (items 4, 9, 11, 12, 13, 20) and stigma resistance–reverse stigma (items 7, 14, 24, 26, 27).

It possesses high internal consistency in persons with severe mental disorders, including psychosis. A mean score for the first four subscales was used, as the fifth subscale assesses not stigma but a reaction to it. The questionnaire lacks a cut-off point, with higher scores indicating greater stigma [19,20]. We further categorized participants with high indications for internalized stigma based on a score greater than 50 points on the total ISMI scale. The Chronbach’s alpha coefficient was 0 0.83. The test–retest reliability coefficients varied from 0.81 to 0.91, indicating substantial agreement. The ICC for the total score was 0.83 and for the two factors it was 0.69 and 0.77 respectively [19].

#### 2.2.2. World Health Organization Quality of Life Assessment-BREF

The World Health Organization Quality of Life Assessment-BREF (WHOQOL-BREF) is a self-reported tool consisting of 26 items that cover the following four domains: physical health, mental health, social relationships and environment. All items are rated using a 5-point Likert scale. According to the WHO guidelines, all scores were transformed to a 1–100 scale [21]. Higher scores indicate a better quality of life. Internal consistency of the Greek validation is satisfactory, with alpha values in the range of α = 0.67–0.81 [22].

#### 2.2.3. Liebowitz Social Anxiety Scale—Self Reported

The Liebowitz Social Anxiety Scale (LSAS-SR) comprises 24 items assessing fear/anxiety and avoidance in different social situations. Each item assesses both variables. Participants are asked to rate their fear/anxiety on a four-point scale (from 0 = none to 3 = severe) (LSAS Anxiety subscale) and avoidance, also on a four-point scale (from 0 = never to 3 = usually) (LSAS Avoidance subscale). The total score represents the sum of the scores obtained on both subscales [23,24]. The Greek translation of the LSAS-SR possesses a very high internal consistency (Cronbach’s alpha: 0.97).

#### 2.2.4. Schedule for the Assessment of Insight—Expanded Version

Insight was assessed using the Schedule for the Assessment of Insight—Expanded version (SAI-E). The SAI-E assesses the recognition of having a mental illness (items 1–6), the ability to re-label symptoms as pathological (items 7–9) and the compliance with treatment (items A and B).

Items 1–6 (A and B) are rated from 0 to 2, and items 7–9 from 1 to 4, with the total score ranging from 0 to 28. Although SAI-E lacks a cut-off point, higher scores indicate better insight [25,26]. The SAI-E is a clinician-administered scale whose Greek validation possesses a high level of internal consistency (0.91) and high test retest reliability (total score 0.88) [26].

#### 2.2.5. Positive and Negative Syndrome Scale

The psychopathology of each participant was estimated using the Positive and Negative Syndrome Scale (PANSS) at two time points (admission to the hospital and six months after discharge). We assessed psychopathology at two time points, assuming that the severity of symptoms, especially of positive symptoms, at the onset of psychosis may impact stigma levels assessed after their remission. Stigma, insight, social anxiety, and quality-of-life measures were administered at the follow-up assessment, six months after clinical stabilization.

We used the Greek translated/validated versions for the above-mentioned scales [19,22,26,27].

Standardized Cronbach’s alpha (0–100) for internal consistency for the scales we used in the present study is as follows:

WHOQOL_BREF: α = 0.86, ISMI: α = 0.91, LSAS: α = 0.98, SAI: α = 0.54.

### 2.3. Ethical Issues

The Ethical Committee of the University Hospital of Ioannina, Greece, approved the protocol. Protocol code: 140/11-04-2019.

### 2.4. Data and Statistical Analysis

To reduce the risk of type I error, analyses were guided by predefined conceptual hypotheses rather than exploratory testing.

Data were collected and analyzed with SPSS version 26.0 (IBM Corp., Armonk, NY, USA). Descriptive statistics were computed; categorical variables were reported as frequencies and percentages, while continuous variables were reported as ranges, means, and standard deviations (SD). The Pearson product–moment correlation coefficient assessed the linear association between the different scales and subscales. Mann–Whitney tests were used to investigate possible differences between genders across all scales. Univariable linear (for ISMI and as WHOQOL-BREF outcomes) or logistic (for high indication of internalized stigma) regression analyses were performed using the following candidate predictor variables: age at entry, gender, marital status, education, medication, children, employment, main residence, psychopathology initially and six months after discharge (PANSS baseline, PANSS at six-month follow-up), duration of untreated psychosis (DUP), insight (SAI-E), social anxiety and avoidance (LSAS Anxiety, LSAS Avoidance, LSAS Total). For WHOQOL-BREF, the ISMI score was further used as a predictor variable, assuming that stigma is more likely to affect one’s quality of life. Multivariable regression models were constructed, adjusting for age, gender, and variables showing potential association in univariable analyses (*p* < 0.20), consistent with established epidemiological modeling approaches. The same prespecified adjustment framework was applied across all outcomes to improve comparability of estimates. Regression assumptions were examined and no major violations were identified.

The study was reported in accordance with STROBE guidelines for observational studies.

## 3. Results

The primary finding was the consistent association between higher internalized stigma and poorer quality of life across all WHOQOL-BREF domains. The sample consisted of 90 patients (55 males and 35 females) with a mean age of 31.8 ± 8.6 years (range: 18–52). Baseline demographic and clinical characteristics are summarized in Table 1 and Table 2. Most participants were diagnosed with schizophreniform disorder (n = 78; 86.7%), and the mean duration of untreated psychosis was 13.4 ± 6.2 weeks. The medications used were risperidone (n = 24; 26.7%), clozapine (n = 34; 37.8%), and olanzapine (n = 32; 35.6%). No statistically significant sex differences were observed in SAI-E, ISMI, WHOQOL-BREF or LSAS scores.

The mean ISMI total score was 50.9 ± 7.9 (range: 29–84), with 56.6% of patients scoring greater than 50, indicative of internalized stigma. Mean PANSS-total scores improved from 76.1 ± 7.2 at baseline to 44.9 ± 6.3 after six months of follow-up (*p* < 0.001). Patients demonstrated insight of (mean ± SD) 20.3 ± 4.5, measured by SAI-E, and moderate-to-good quality of life across WHOQOL-BREF domains (Table 3).

Supplementary Table S1 summarizes the correlations among insight, internalized stigma, quality of life and social anxiety measures. Overall, internalized stigma demonstrated the strongest and most consistent associations across the examined psychosocial measures.

Higher ISMI total scores were moderately to strongly associated with poorer quality of life across all WHOQOL-BREF domains (r = −0.47 to −0.61, all *p* < 0.001). ISMI was also positively associated with social anxiety and avoidance, showing moderate correlations with LSAS Total scores (r = 0.41, *p* < 0.001). Similar patterns were observed for most ISMI subscales (Supplementary Table S1).

No significant correlation was observed between the total SAI-E and ISMI scores. However, selected SAI-E subscales showed modest correlations with ISMI sub-domains, particularly the “Recognition of having a mental illness” subscale, which was positively associated with several dimensions of internalized stigma (Supplementary Table S1).

The SAI-E total score showed weak negative correlations with the WHOQOL-BREF physical health (r = −0.278, *p* = 0.008) and mental health (r = −0.234, *p* = 0.001) domains, whereas treatment compliance was weakly associated with better mental health (r = 0.208, *p* = 0.049) and environment scores (r = 0.254, *p* = 0.016).

Regarding predictors of internalized stigma for mental illness, in the univariable models, employment status (*p* = 0.037), LSAS-SR Total (*p* < 0.001), and SAI-E (*p* = 0.064) were associated with the ISMI score. LSAS-SR Total remained statistically significant with positive association with the ISMI score in the multivariable model (Beta = 0.18, 95% confidence interval [CI]: 0.08 to 0.28, *p* < 0.001) (Supplementary Table S2). We further categorized the ISMI score to high (>50) or low (≤50) levels as an exploratory and descriptive secondary analysis to identify participants with higher internalized stigma in this sample. If indication of internalized stigma, medication, SAI-E, and LSAS-SR Total were identified as possible predictors for the univariable models, in the multivariable models only medication remained statistically significant (odds ratio [OR] for risperidone vs clozapine = 0.12, 95% CI: 0.03 to 0.48, *p* = 0.003), while there was an indication of a positive association with SAI-E and LSAS-SR Total (Supplementary Table S3).

As for predictors of quality of life, although several demographic and clinical variables were associated with individual WHOQOL-BREF domains in the univariable analyses (Table 4), none remained independently associated after adjustment. In multivariable analyses, internalized stigma emerged as the only independent predictor consistently associated with all four WHOQOL-BREF domains. One unit increase in the ISMI scale was independently associated with −0.73 units in WHOQOL-BREF physical health (95% CI: −1.09, −0.36), −1.03 units in WHOQOL BREF mental health (95% CI: −1.44, −0.62), −0.78 units in WHOQOL-BREF social relationships (95% CI: −1.38, −0.18), and −0.68 units in WHOQOL-BREF environment (95% CI: −0.99, −0.38) (Table 5).

## 4. Discussion

In this study, internalized stigma was reported by 56.6% of participants, with 36.6% demonstrating moderate-to-high stigma levels (ISMI > 60) and 1.1% very high stigma severity (ISMI > 70). Ho et al. (2018) [12] reported that, in a sample of 136 persons with FEP, 36.8% had moderate-to-high self-stigma levels at 3-year follow-up. Together, these outcomes indicate that internalized stigma is already well established early in the course of psychosis rather than emerging solely as a consequence of chronic illness.

Internalized stigma was strongly associated with poorer quality of life across domains. Wang et al. (2016) [28] report in a sample of 128 Chinese patients with schizophrenia a strong reduction in quality of life in the domains of psychosocial and motivation/energy one month after discharge from the hospital. Park et al. (2013) [29] report that higher internalized stigma is correlated with poorer quality of life in the Satisfaction with Family and Satisfaction with Social Relations subscales, but not in the Family Contact and Social Contact subscales. They conclude that internalized stigma affects satisfaction, yet not the quantity of contact. Higher insight levels were associated with increased odds of elevated internalized stigma, while greater stigma independently predicted poorer quality of life after adjustment for covariates.

Chio et al. (2018) [30] report that good insight at baseline was correlated with lower life satisfaction at 6-month follow-up when self-stigma was high, but with better quality of life at one-year follow-up when self-stigma was low. These outcomes indicate that recovery processes may be adversely affected when increased illness awareness is not accompanied by reductions in self-stigma. Buchman-Wildbaum et al. (2020) [31] report in a sample of 200 patients with mental illness (53 of them having a diagnosis of schizophrenia) that better insight was correlated with higher internalized stigma, lower self-esteem and increased experience of shame. These data support the hypothesis of an “insight paradox” [32], namely that having good insight into one’s illness may also have negative or even detrimental consequences. Our findings support the “insight paradox” framework, whereby increased illness awareness may coexist with increased self-stigmatization, possibly compromising subjective recovery despite clinical stabilization. Previous research has linked higher levels of insight with depression, anxiety, poorer quality of life, and reduced self-esteem [31]. Chu et al. (2023) [16] report that higher levels of insight were associated with higher self-stigma levels. In this study, the SAI-E subscale “recognition of having a mental illness” was positively correlated with the total ISMI scale and the subscales “Alienation”, “Discrimination experience” and “Social withdrawal” while the SAI-E subscale “ability to re-label symptoms as pathological” was negatively associated with the ISMI subscale “stereotype endorsement”.

In our study, social anxiety and avoidance closely paralleled internalized stigma levels. In a study conducted by Brain et al. (2014) [33], the authors report that more than half of the participants felt socially excluded. The most common coping strategy they used to bypass stigma and discrimination was avoidance. Almost all concealed diagnoses, avoided close relationships and did not apply for a job. Lysaker et al. (2007) [32] and Park et al. (2013) [29] did not find any correlations between negative symptoms and stigma levels in individuals with chronic schizophrenia, while Rector et al. (2005) [34] report an association between internalized stigma and negative symptoms. In this study, we did not find any correlation between negative symptoms, either at baseline or at six-month follow-up.

Baseline positive symptoms demonstrated a small but significant association with internalized stigma, suggesting that acute symptom expression may raise vulnerability to stigmatizing experiences. Ho et al. (2018) [12] report that more severe positive symptoms at intake predicted higher self-stigma levels at follow-up, and Tarrier et al. (2007) [35] found that the severity of positive symptoms is associated with higher perceived stigma. More severe positive symptoms may lead to agreement with stereotypes that persons with psychosis are unpredictable, unreliable, aggressive or peculiar and to internalization of social stigma. In the study by Simonsen et al. (2019) [13] in a sample of 112 participants, the sustainability of stigma after one year of treatment was associated with poorer clinical outcome in terms of symptoms, functioning and disability and life satisfaction. On the contrary, Chu et al. (2023) [16] did not find any statistically significant association between self-stigma and positive, negative or depressive symptoms in a sample of 101 FEP patients.

Furthermore, Ho et al. (2018) [12] report a significant positive correlation between higher stigma levels and longer DUP and suggest that longer exposure to public stigma may lead to increased internalized stigma. Chu et al. (2023) [16] also report greater self-stigma in FEP patients with longer DUP while Roldua-Ros et al. (2025) [36] found that DUP was the only significant predictor for the social isolation subscale of the ISMI scale. The authors suggest that a shorter DUP may be associated with reduced social isolation and better quality of life. No association between DUP and internalized stigma was observed in the present sample. The absence of association with DUP may reflect the relatively short untreated duration in this cohort, limiting exposure to public stigma during early illness stages.

Finally, the results of the present study suggest that stigma experiences are not associated with gender. Ho et al. (2018) [12] report an association between female gender and higher stigma levels in first-episode patients, and Jerkins and Carpenter-Song (2009) [9] report an association between female gender and higher stigma levels in a sample of chronic schizophrenia patients and a recent study by Chu et al. (2023) [16] reports higher self-stigma levels in females with FEP than in males. On the contrary, Livingston & Boyd (2010) [37], in a meta-analysis conducted in a sample of persons with chronic psychiatric disorders, did not find gender differences in stigma levels.

## 5. Limitations

Despite these clinically meaningful findings, several methodological limitations should be acknowledged. Participants were recruited from a single specialized early-intervention service; therefore, findings may primarily generalize to patients receiving care within structured early-intervention services. The cross-sectional design precludes causal inference regarding the relationship between stigma and psychosocial outcomes. The relatively modest sample size from a single early-intervention service may limit generalizability. The categorization of ISMI using 50 points as a cut-off was conducted as an exploratory and descriptive secondary analysis to identify participants with higher internalized stigma in this sample. This threshold is not a validated diagnostic cut-off and results based on this categorization should be interpreted cautiously. Moreover, several outcomes, particularly WHOQOL-BREF domains, were correlated; therefore, findings should be interpreted according to the consistency and direction of associations rather than in isolation. The modest sample size also increased the risk of overfitting in multivariable models, especially logistic regression analyses, and these results should be interpreted with caution.

Self-report measures may introduce response bias, even when validated instruments are used. Finally, longitudinal studies are needed to clarify the temporal relationships among insight, stigma, and recovery outcomes.

Although the sample size was limited and some analyses were likely underpowered, the findings from the univariable and multivariable analyses were generally consistent. Furthermore, the small sample limitation should be interpreted in the context of the challenges associated with recruiting this specific study population, which is relatively difficult to access and enroll.

Medication-related findings should also be interpreted cautiously, as treatment allocation may have been influenced by clinical severity, indication, tolerability, treatment resistance, and service-level decision-making.

### Future Research Directions

Longitudinal designs and anonymized datasets from different sites could provide more generalizable results while psychosocial interventions could minimize internalized stigma and also public stigma—probably the source of internalized stigma—and their disastrous consequences.

## 6. Conclusions

The study’s results indicated that stigma as assessed by the Internalized Stigma for Mental Illness Scale is positively correlated with social anxiety. Higher stigma levels were independently linearly associated with lower levels of quality of life, as expressed in all WHOQOL-BREF assessment domains (physical health, mental health, social relationships, environment), even after adjusting for other predictor variables. Our results also indicated an association between higher insight levels and elevated odds of high internalized stigma.

## Figures and Tables

**Table 1:** Sample-group (n = 90) baseline characteristics.

		N (%)
Gender	Male	55 (61.1)
	Female	35 (38.9)
Education	Up to high school	41 (45.6)
	Higher than high school	49 (54.4)
Marital status	Single	72 (80.0)
	Married	11 (12.2)
	Divorced	7 (7.8)
Employment	Unemployed	64 (71.1)
	Part-time job	6 (6.7)
	Full-time job	18 (20.0)
	Practice	1 (1.1)
	Sickness leave	1 (1.1)
Residence in an area with	<10.000 inhabitants	15 (16.7)
	10.000> and <100.000 inhabitants	10 (11.1)
	>100.000 inhabitants	65 (72.2)
Diagnosis	Brief psychotic episode	8 (8.9)
	Schizophreniform disorder	78 (86.7)
	Schizophrenia	4 (4.4)
Medication Type	Risperidone	24 (26.7)
	Clozapine	34 (37.8)
	Olanzapine	32 (35.6)

**Table 2:** Sample-group psychopathology as assessed by the PANSS at baseline and at six-month follow-up.

	Total (n = 90)		Males (n = 55)		Females (n = 35)		Gender Differences *p*-Value
Variable	Mean ± SD	Min, Max	Mean ± SD	Min, Max	Mean ± SD	Min, Max	
Baseline							
PANSS-p	31.6 ± 2.8	27 to 38	32.0 ± 3.0	27 to 38	31.0 ± 2.4	28 to 36	0.093
PANSS-n	21.1 ± 3.5	14 to 29	21.8 ± 3.5	14 to 29	20.0 ± 3.3	14 to 27	0.024
PANSS-g	23.4 ± 2.4	18 to 28	23.6 ± 2.5	18 to 28	23.1 ± 2.3	19 to 27	0.271
PANSS-t	76.1 ± 7.2	62 to 93	77.4 ± 7.4	63 to 93	74.1 ± 6.5	62 to 87	0.028
DUP (weeks)	13.4 ± 6.2	2 to 28	14.5 ± 6.2	4 to 28	11.7 ± 6.0	2 to 23	0.050
6-month follow-up							
PANSS-p	7.7 ± 1.0	7 to 10	7.7 ± 1.0	7 to 10	7.7 ± 1.0	7 to 10	0.634
PANSS-n	18.2 ± 3.4	11 to 28	18.7 ± 3.5	12 to 28	17.4 ± 3.0	11 to 23	0.092
PANSS-g	19.1 ± 2.8	13 to 25	19.5 ± 2.9	14 to 25	18.5 ± 2.7	13 to 24	0.124
PANSS-t	44.9 ± 6.3	31 to 57	45.7 ± 6.5	31 to 57	43.5 ± 5.8	32 to 56	0.099

Abbreviations: DUP: duration of untreated psychosis; PANSS: Positive and Negative Syndrome Scale (PANSS-p: positive, PANSS-n: negative, PANSS-g: general psychopathology, PANSS-t: total score); SD: standard deviation.

**Table 3:** Sample-group (n = 90) scores on SAI-E, WHOQOL-BREF, LSAS-SR, and ISMI scales and subscales at six-month follow-up.

	Total (n = 90)		Males (n = 55)		Females (n = 35)		Gender Differences *p*-Value
Variable	Mean ± SD	Min, Max	Mean ± SD	Min, Max	Mean ± SD	Min, Max	
SAI-E							
Recognition of mental illness	7.6 ± 2.6	0 to 12	7.5 ± 2.5	2 to 12	7.7 ± 2.7	0 to 12	0.520
Ability to re-label symptoms as abnormal	9.1 ± 2.3	3 to 12	9.3 ± 2.2	5 to 12	8.7 ± 2.4	3 to 12	0.250
Compliance with treatment	3.7 ± 0.8	0 to 4	3.7 ± 0.7	2 to 4	3.7 ± 1.0	0 to 4	0.308
Total score	20.3 ± 4.4	3 to 28	20.5 ± 4.1	12 to 28	20.1 ± 5.0	3 to 28	0.993
WHOQOL-BREF							
Physical health	71.6 ± 13.4	38 to 100	69.7 ± 13.7	38 to 100	74.6 ± 12.7	38 to 100	0.054
Mental health	71.4 ± 15.7	31 to 100	72.9 ± 15.7	38 to 100	69.2 ± 15.8	31 to 94	0.356
Social relationships	56.9 ± 21.5	0 to 100	56.7 ± 21.6	0 to 100	57.2 ± 21.8	25 to 100	0.907
Environment	73.5 ± 11.2	44 to 94	73.7 ± 10.8	44 to 94	73.2 ± 12	44 to 94	0.977
LSAS							
Anxiety	3.3 ± 8.7	0 to 44	3.0 ± 8.7	0 to 44	3.7 ± 8.6	0 to 39	0.865
Avoidance	2.9 ± 7.6	0 to 44	2.7 ± 7.9	0 to 44	3.3 ± 7.3	0 to 28	0.741
Total	6.2 ± 16.2	0 to 88	5.7 ± 16.5	0 to 88	7.0 ± 15.8	0 to 65	0.854
ISMI							
Alienation	12.9 ± 2.7	7 to 22	12.8 ± 2.6	7 to 19	12.9 ± 2.9	7 to 22	0.792
Stereotype endorsement	14.4 ± 2.3	7 to 22	14.2 ± 2.3	7 to 20	14.6 ± 2.4	9 to 22	0.815
Discrimination experience	11.2 ± 2.2	5 to 18	10.9 ± 2.2	5 to 16	11.7 ± 2.1	9 to 18	0.142
Social withdrawal	12.5 ± 2.5	6 to 22	12.3 ± 2.4	6 to 18	12.9 ± 2.6	7 to 22	0.605
Stigma resistance	12.7 ± 1.8	8 to 18	12.8 ± 1.9	8 to 18	12.6 ± 1.7	8 to 15	0.688
Total score	50.9 ± 7.9	29 to 84	50.2 ± 7.5	29 to 66	52.1 ± 8.3	36 to 84	0.321

Abbreviations: ISMI: Internalized Stigma for Mental Illness; LSAS: Liebowitz Social Anxiety Scale; SAI-E: Schedule for the Assessment of Insight—Expanded version; SD: Standard deviation; WHOQOL-BREF: World Health Organization Quality of Life Assessment.

**Table 4:** Univariable linear regression analyses for the association of the candidate predictor variables with World Health Organization Quality of Life Assessment.

	Physical Health		Mental Health		Social Relationships		Environmental Health	
Variable	Beta (95% CI)	*p*-Value	Beta (95% CI)	*p*-Value	Beta (95% CI)	*p*-Value	Beta (95% CI)	*p*-Value
Age (per year)	−0.13 (−0.46, 0.20)	0.429	0.05 (−0.34, 0.43)	0.813	0.25 (−0.28, 0.77)	0.353	−0.08 (−0.36, 0.19)	0.543
Gender								
Male	Reference		Reference		Reference		Reference	
Female	4.90 (−0.81, 10.61)	0.092 *	−3.72 (−10.47, 3.03)	0.277	0.48 (−8.82, 9.78)	0.918	−0.52 (−5.37, 4.33)	0.832
Marital status								
Unmarried	Reference		Reference		Reference		Reference	
Married	−1.72 (−10.45, 7.01)	0.696	1.59 (−8.59, 11.78)	0.757	22.30 (9.17, 35.42)	0.001 ***	−2.40 (−9.65, 4.86)	0.513
Divorced	1.56 (−9.11, 12.24)	0.771	−5.54 (−17.99, 6.92)	0.379	−3.57 (−19.63, 12.48)	0.659	−4.41 (−13.28, 4.46)	0.326
Education								
Up to high school	Reference		Reference		Reference		Reference	
Higher than high school	4.43 (−1.17, 10.04)	0.120	3.37 (−3.25, 9.99)	0.314	0.00 (−9.10, 9.10)	1.000	3.63 (−1.06, 8.32)	0.127
Medication type								
Clozapine	Reference		Reference		Reference		Reference	
Olanzapine	−2.94 (−10.09, 4.22)	0.417	−4.12 (−12.46, 4.21)	0.328	4.03 (−7.26, 15.32)	0.480	2.14 (−3.66, 7.94)	0.465
Risperidone	0.45 (−6.80, 7.69)	0.902	1.06 (−7.38, 9.51)	0.803	10.84 (−0.59, 22.28)	0.063 *	7.19 (1.31, 13.06)	0.017 **
Has children								
No	Reference		Reference		Reference		Reference	
Yes	−1.32 (−8.91, 6.27)	0.730	1.31 (−7.58, 10.20)	0.771	11.27 (−0.66, 23.20)	0.064 *	−3.55 (−9.85, 2.76)	0.266
Employment								
No	Reference		Reference		Reference	Reference	Reference	
Yes	3.60 (−2.60, 9.80)	0.252	6.89 (−0.27, 14.06)	0.059 *	7.91 (−1.96, 17.77)	0.115	0.00 (0.00, 0.00)	0.310
Residence in an area with								
<10.000 inhabitants	Reference		Reference		Reference		Reference	
10.000> and <100.000 inhabitants	3.60 (−7.39, 14.59)	0.517	1.97 (−10.94, 14.87)	0.767	17.33 (0.08, 34.59)	0.049 **	8.03 (−0.73, 16.80)	0.072 *
>100.000 inhabitants	0.32 (−7.39, 8.04)	0.934	0.96 (−8.10, 10.01)	0.834	9.26 (−2.85, 21.36)	0.132	9.32 (3.17, 15.47)	0.003 **
PANSS-T (baseline) (per unit)	0.03 (−0.37, 0.43)	0.874	0.31 (−0.15, 0.77)	0.183	−0.41 (−1.04, 0.22)	0.197	−0.08 (−0.41, 0.25)	0.620
PANSS-T (final) (per unit)	−0.08 (−0.53, 0.38)	0.736	−0.15 (−0.68, 0.38)	0.581	−0.18 (−0.91, 0.54)	0.615	−0.09 (−0.47, 0.29)	0.638
DUP (per week)	0.22 (−0.23, 0.68)	0.337	−0.22 (−0.76, 0.31)	0.407	−0.03 (−0.76, 0.70)	0.936	−0.39 (−0.77, −0.02)	0.039 **
SAI-E (per unit)	−0.84 (−1.45, −0.23)	0.008 ***	−0.83 (−1.55, −0.10)	0.027 **	0.01 (−1.02, 1.03)	0.991	−0.08 (−0.61, 0.46)	0.774
ISMI (per unit)	−0.80 (−1.12, −0.48)	<0.001 ***	−1.21 (−1.55, −0.88)	<0.001 ***	−1.11 (−1.64, −0.58)	<0.001 ***	−0.67 (−0.94, −0.40)	<0.001 ***
LSAS-SR Total (per unit)	−0.23 (−0.40, −0.06)	0.009 ***	−0.36 (−0.56, −0.17)	<0.001 ***	−0.44 (−0.71, −0.17)	0.001 ***	−0.09 (−0.24, 0.05)	0.209

Legend: * *p* < 0.10, ** *p* < 0.05, *** *p* < 0.01. Abbreviations: CI: confidence interval; DUP: duration of untreated psychosis; ISMI: Internalized Stigma for Mental Illness; LSAS-SR: Liebowitz Social Anxiety Scale; PANSS: Positive and Negative Syndrome Scale—total score; SAI-E: Schedule for the Assessment of Insight—Expanded version.

**Table 5:** Multivariable linear regression analyses for the association of the candidate predictor variables with World Health Organization Quality of Life Assessment—Greek version.

	Physical Health		Mental Health		Social Relationships		Environmental Health	
Variable	Beta (95% CI)	*p*-Value	Beta (95% CI)	*p*-Value	Beta (95% CI)	*p*-Value	Beta (95% CI)	*p*-Value
Age (per year)	−0.20 (−0.57, 0.17)	0.275	0.01 (−0.40, 0.42)	0.960	−0.07 (−0.67, 0.54)	0.827	−0.03 (−0.34, 0.27)	0.835
Gender								
Male	Reference		Reference		Reference		Reference	
Female	6.63 (1.09, 12.17)	0.020 **	−1.77 (−7.97, 4.44)	0.572	0.56 (−8.57, 9.69)	0.903	−0.69 (−5.29, 3.92)	0.767
Marital status								
Unmarried	Reference		Reference		Reference		Reference	
Married	−0.57 (−12.36, 11.22)	0.923	−5.22 (−18.43, 7.99)	0.434	16.30 (−3.14, 35.73)	0.099 *	−1.53 (−11.32, 8.27)	0.757
Divorced	8.02 (−6.48, 22.53)	0.274	−6.96 (−23.20, 9.29)	0.397	−5.36 (−29.27, 18.54)	0.656	1.88 (−10.16, 13.92)	0.757
Medication type								
Clozapine	Reference		Reference		Reference		Reference	
Olanzapine	0.53 (−6.46, 7.51)	0.881	−0.98 (−8.81, 6.84)	0.803	6.49 (−5.02, 18.01)	0.265	2.78 (−3.02, 8.58)	0.343
Risperidone	0.22 (−6.78, 7.23)	0.949	−0.20 (−8.05, 7.66)	0.961	11.41 (−0.14, 22.96)	0.053 *	4.50 (−1.32, 10.32)	0.128
Has children								
No	Reference		Reference		Reference		Reference	
Yes	−4.40 (−17.03, 8.22)	0.489	5.10 (−9.04, 19.25)	0.475	4.34 (−16.47, 25.16)	0.679	−2.68 (−13.17, 7.80)	0.612
Employment								
No	Reference		Reference		Reference		Reference	
Yes	1.76 (−4.23, 7.74)	0.560	1.54 (−5.17, 8.24)	0.649	−0.22 (−10.08, 9.65)	0.965	0.33 (−4.64, 5.30)	0.895
Residence in an area with								
<10.000 inhabitants	Reference		Reference		Reference		Reference	
10.000> and <100.000 inhabitants	2.99 (−8.40, 14.38)	0.602	−1.10 (−13.86, 11.66)	0.864	2.34 (−16.44, 21.11)	0.805	4.33 (−5.13, 13.79)	0.365
>100.000 inhabitants	−0.18 (−7.40, 7.05)	0.961	−1.11 (−9.20, 6.99)	0.786	4.18 (−7.73, 16.09)	0.486	6.40 (0.40, 12.40)	0.037 **
DUP (per week)	0.35 (−0.08, 0.78)	0.105	−0.31 (−0.79, 0.17)	0.197	0.07 (−0.64, 0.77)	0.852	−0.37 (−0.72, −0.01)	0.044 **
SAI-E (per unit)	−0.64 (−1.26, 0.02)	0.043 **	−0.27 (−0.96, 0.43)	0.443	0.23 (−0.79, 1.25)	0.654	0.12 (−0.40, 0.63)	0.654
ISMI (per unit)	−0.73 (−1.09, −0.36)	<0.001 ***	−1.03 (−1.44, −0.62)	<0.001 ***	−0.78 (−1.38, −0.18)	0.012 ***	−0.68 (−0.99, −0.38)	<0.001 ***
LSAS-SR Total (per unit)	0.07 (−0.24, 0.11)	0.435	−0.15 (−0.34, 0.05)	0.133	−0.27 (−0.56, 0.02)	0.067 *	0.01 (−0.13, 0.16)	0.871

Legend: * *p* < 0.10, ** *p* < 0.05, *** *p* < 0.01. Abbreviations: CI: confidence interval; DUP: duration of untreated psychosis; ISMI: Internalized Stigma for Mental Illness; LSAS-SR: Liebowitz Social Anxiety Scale; SAI-E: Schedule for the Assessment of Insight—Expanded version.

## Data Availability

The data supporting the findings of this study are not publicly available due to ethical and privacy restrictions involving sensitive participant information. Further inquiries may be directed to the corresponding author.

## Supplementary Materials

The following supporting information can be downloaded at: https://www.mdpi.com/article/10.3390/healthcare14152342/s1, Table S1: Correlation between the ISMI, LSAS, SAI-E, and WHOQOL-BREF scores.; Table S2: Univariable and multivariable linear regression analyses for the association of the candidate predictor variables with high the total Internalized Stigma for Mental Illness Scale (ISMI) scale.; Table S3: Univariable and multivariable logistic regression analyses for the association of the candidate predictor variables with high indication of internalized stigma, based on a score higher than 50 points in the total Internalized Stigma for Mental Illness Scale (ISMI) scale.
