# Supplementary material for: Internalized Stigma, Insight, Social Anxiety and Quality of Life in First-Episode Psychosis: A Cross-Sectional Study
Source: Healthcare (Basel). 2026 Aug 1;14(15):2342. doi: 10.3390/healthcare14152342 (PMC13465740; doi:10.3390/healthcare14152342)
Supplement: Supplementary file 1 [file healthcare-14-02342-s001.zip › healthcare-4412220-supplementary.pdf]

Supplementary Table S1: Correlation between the ISMI, LSAS, SAI-E, and WHOQOL-BREF scores.

		SAI-E				WHOQOL-BREF				LSAS			ISMI				
		Recognition Mental Illness	Ability relabel symptom abnormal	Compliance with treatment	**Total score**	Physical health and level of independence	Mental health and spirituality	Social relationships	Environmental health	**Anxiety**	**Avoidance**	**Total**	Alienation	Stereotype endorsement	Discrimination experience	Social withdrawal	Stigma resistance
**SAI-E**	Ability relabel symptom abnormal	0.375***	1														
		<0.001	-														
	Compliance with treatment	0.413***	0.282***	1													
		<0.001	0.007	-													
	**Total score**	0.849***	0.783***	0.569***	1												
		<0.001	<0.001	<0.001	-												
**WHOQOL-BREF**	Physical health-level of independence	-0.422***	-0.047	-0.048	-0.278***	1											
		<0.001	0.657	0.654	0.008	-											
	Mental health and spirituality	-0.390***	-0.089	0.208**	-0.234**	0.544***	1										
		<0.001	0.404	0.049	0.027	<0.001	-										
	Social relationships	-0.166	0.124	0.182*	0.001	0.348***	0.476***	1									
		0.119	0.243	0.086	0.991	0.001	<0.001	-									
	Environmental health	-0.251**	0.133	0.254**	-0.031	0.392***	0.615***	0.450***	1								
		0.017	0.211	0.016	0.774	<0.001	<0.001	<0.001	-								
**LSAS**	**Anxiety**	0.260**	-0.085	-0.064	0.096	-0.264**	-0.371***	-0.326***	-0.131	1							
		0.013	0.426	0.550	0.369	0.012	<0.001	0.002	0.220	-							
	**Avoidance**	0.278***	-0.059	-0.011	0.129	-0.281***	-0.374***	-0.334***	-0.136	0.981***	1						
		0.008	0.579	0.919	0.224	0.007	<0.001	0.001	0.202	<0.001	-						
	**Total**	0.270**	-0.073	-0.039	0.112	-0.273***	-0.374***	-0.331***	-0.134	0.996***	0.995***	1					
		0.010	0.493	0.714	0.293	0.009	<0.001	0.001	0.209	<0.001	<0.001	-					
**ISMI**	Alienation	0.351***	0.060	-0.002	0.235**	-0.430***	-0.540***	-0.194*	-0.348***	0.216**	0.252**	0.234**	1				
		0.001	0.572	0.987	0.026	<0.001	<0.001	0.066	0.001	0.041	0.017	0.026	-				
	Stereotype endorsement	0.206*	-0.250**	-0.114	-0.030	-0.238**	-0.343***	-0.341***	-0.354***	0.299***	0.299***	0.300***	0.421***	1			
		0.052	0.017	0.285	0.778	0.024	0.001	0.001	0.001	0.004	0.004	0.004	<0.001	-			
	Discrimination experience	0.295***	-0.047	-0.008	0.146	-0.394***	-0.557***	-0.473***	-0.377***	0.476***	0.480***	0.480***	0.555***	0.587***	1		
		0.005	0.658	0.937	0.171	<0.001	<0.001	<0.001	<0.001	<0.001	<0.001	<0.001	<0.001	<0.001	-		
	Social withdrawal	0.402***	0.074	-0.028	0.266**	-0.441***	-0.522***	-0.341***	-0.449***	0.334***	0.361***	0.348***	0.570***	0.516***	0.640***	1	
		<0.001	0.488	0.796	0.011	<0.001	<0.001	0.001	<0.001	0.001	<0.001	0.001	<0.001	<0.001	<0.001	-	
	Stigma resistance	-0.006	0.045	0.189*	0.054	0.135	0.324***	0.275***	0.219**	-0.168	-0.141	-0.156	-0.026	-0.242**	-0.301***	0.035	1
		0.957	0.677	0.074	0.611	0.205	0.002	0.009	0.038	0.114	0.185	0.142	0.806	0.022	0.004	0.746	-
	**Total score**	0.390***	-0.043	-0.045	0.196*	-0.466***	-0.606***	-0.406***	-0.470***	0.399***	0.421***	0.411***	0.802***	0.765***	0.842***	0.841***	-0.152
		<0.001	0.688	0.672	0.064	<0.001	<0.001	<0.001	<0.001	<0.001	<0.001	<0.001	<0.001	<0.001	<0.001	<0.001	0.152

Legend: * p<0.10, ** p<0.05, *** p<0.01.

Abbreviations: ISMI: Internalized stigma for mental illness, LSAS: Liebowitz social anxiety scale – self report , SAI-E: Schedule for the assessment of insight - expanded version, WHOQOL BREF: World Health Organization quality of life assessment - Greek version.

Supplementary Table S2: Univariable and multivariable linear regression analyses for the association of the candidate predictor variables with high the total Internalized Stigma for Mental Illness Scale (ISMI) scale.

	Univariable model		Multivariable model	
**Variable**	**Beta (95% CI)**	**p-value**	**Beta (95% CI)**	**p-value**
**Age (per year)**	-0.04 (-0.23, 0.16)	0.706	0.03 (-0.15, 0.20)	0.777
**Gender**				
Male	Reference		Reference	
Female	1.93 (-1.44, 5.30)	0.257	1.51 (-1.61, 4.63)	0.339
**Marital status**				
Unmarried	Reference			
Married	-2.29 (-7.35, 2.78)			
Divorced	2.56 (-3.64, 8.75)	0.440		
**Education**				
Up to High school	Ref			
Higher than High school	-1.06 (-4.38, 2.25)	0.525		
**Medication Type**				
Clozapine	Reference			
Olanzapine	2.67 (-1.45, 6.80)			
Risperidone	-0.80 (-4.98, 3.38)	0.174		
**Has children**				
No	Reference			
Yes	-0.56 (-5.00, 3.88)	0.803		
**Employment**				
No	Reference		Reference	
Yes	-3.80 (-7.36, -0.24)	0.037**	-1.98 (-5.44, 1.49)	0.259
**Residence in an area with**				
< 10.000 inhabitants	Reference			
10.000> and <100.000 inhabitants	-2.67 (-9.07, 3.74)			
>100.000 inhabitants	-2.27 (-6.76, 2.23)	0.578		
**PANSS T (baseline) (per unit)**	-0.07 (-0.30, 0.16)	0.556		
**PANSS T (final) (per unit)**	-0.02 (-0.28, 0.25)	0.907		
**DUP (per week)**	-0.07 (-0.34, 0.20)	0.609		
**SAI E (per unit)**	0.35 (-0.02, 0.71)	0.064*	0.26 (-0.08, 0.60)	0.133
**LSAS SR Total (per unit)**	0.20 (0.11, 0.29)	<0.001***	0.18 (0.08, 0.28)	<0.001***

Legend: * p<0.10, ** p<0.05, *** p<0.01.
Abbreviations:CI: Confidence interval, DUP: Duration of untreated psychosis, ISMI: Internalized stigma for mental illness, LSAS: Liebowitz social anxiety scale, PANSS: Positive and Negative Syndrome Scale - total score, SAI-E: Schedule for the assessment of insight - expanded version.

Supplementary Table S3: Univariable and multivariable logistic regression analyses for the association of the candidate predictor variables with high indication of Internalized Stigma, based on a score larger than 50 points in the total Internalized Stigma for Mental Illness Scale (ISMI) scale.

	Univariable model		Multivariable model	
**Variable**	**OR (95% CI)**	**p-value**	**OR (95% CI)**	**p-value**
**Age (per year)**	1.00 (0.96, 1.05)	0.928	1.00 (0.94, 1.07)	0.918
**Gender**				
Male	Reference		Reference	
Female	1.25 (0.53, 2.95)	0.610	1.59 (0.51, 4.91)	0.425
**Marital status**				
Unmarried	Reference			
Married	0.67 (0.19, 2.39)			
Divorced	4.80 (0.55, 41.93)	0.188		
**Education**				
Up to High school	Reference			
Higher than High school	0.60 (0.26, 1.40)	0.236		
**Medication Type**				
Clozapine	Reference		Reference	
Olanzapine	1.95 (0.62, 6.13)		1.28 (0.33, 4.95)	
Risperidone	0.27 (0.09, 0.83)	0.001***	0.12 (0.03, 0.48)	0.003***
**Has children**				
No	Reference			
Yes	1.18 (0.38, 3.65)	0.775		
**Employment**				
No	Reference			
Yes	0.55 (0.22, 1.38)	0.201		
**Residence in an area with**				
< 10.000 inhabitants	Reference			
10.000> and <100.000 inhabitants	0.21 (0.04, 1.21)			
>100.000 inhabitants	0.70 (0.22, 2.29)	0.164		
**PANSS T (baseline) (per unit)**	1.02 (0.96, 1.08)	0.542		
**PANSS T (final) (per unit)**	1.03 (0.96, 1.10)	0.448		
**DUP (per week)**	1.01 (0.94, 1.08)	0.809		
**SAI E (per unit)**	1.09 (0.99, 1.20)	0.091*	1.12 (0.99, 1.27)	0.067*
**LSAS SR Total (per unit)**	1.16 (1.00, 1.35)	0.048**	1.14 (1.00, 1.30)	0.051*

Legend: * p<0.10, ** p<0.05, *** p<0.01.

Abbreviations:CI: Confidence interval, DUP: Duration of untreated psychosis, ISMI: Internalized stigma for mental illness, LSAS-SR-Gr: Liebowitz social anxiety scale – self report - Greek version, PANSS: Positive and Negative Syndrome Scale - total score, SAI-E: Schedule for the assessment of insight - expanded version.
